# Identifying frequent patterns in biochemical reaction networks: a workflow

**DOI:** 10.1093/database/bay051

**Published:** 2018-07-03

**Authors:** Fabienne Lambusch, Dagmar Waltemath, Olaf Wolkenhauer, Kurt Sandkuhl, Christian Rosenke, Ron Henkel

**Affiliations:** 1 Business Information Systems, University of Rostock, Rostock, Mecklenburg-Vorpommern, Germany; 2 Department of Systems Biology and Bioinformatics, University of Rostock, Rostock, Mecklenburg-Vorpommern, Germany; 3 Stellenbosch Institute for Advanced Study (STIAS), Wallenberg Research Centre, Stellenbosch University, Stellenbosch, South Africa; 4 ITMO University, 49 Kronverksky Pr., St. Petersburg, Russia; 5 Visual Computing and Computer Graphics, University of Rostock, Rostock, Mecklenburg-Vorpommern, Germany; 6 Scientific Databases and Visualization, Heidelberg Institute for Theoretical Studies, Heidelberg, Germany

## Abstract

Computational models in biology encode molecular and cell biological processes. Many of these models can be represented as biochemical reaction networks. Studying such networks, one is mostly interested in systems that share similar reactions and mechanisms. Typical goals of an investigation thus include understanding of model parts, identification of reoccurring patterns and recognition of biologically relevant motifs. The large number and size of available models, however, require automated methods to support researchers in achieving their goals. Specifically for the problem of finding patterns in large networks only partial solutions exist. We propose a workflow that identifies frequent structural patterns in biochemical reaction networks encoded in the Systems Biology Markup Language. The workflow utilizes a subgraph mining algorithm to detect the network patterns. Once patterns are identified, the textual pattern description can automatically be converted into a graphical representation. Furthermore, information about the distribution of patterns among a selected set of models can be retrieved. The workflow was validated with 575 models from the curated branch of BioModels. In this paper, we highlight interesting and frequent structural patterns. Furthermore, we provide exemplary patterns that incorporate terms from the Systems Biology Ontology. Our workflow can be applied to a custom set of models or to models already existing in our graph database MaSyMoS. The occurrences of frequent patterns may give insight into the encoding of central biological processes, evaluate postulated biological motifs or serve as a similarity measure for models that share common structures.

Database URL: https://github.com/FabienneL/BioNet-Mining

## Introduction

Modelling is an integral part of computational biology (1). Its increasing impact is reflected in the rapidly growing number and complexity of computational models (2, 3). Such models encode a wide range of biological processes [including cell cycle processes, apoptosis, mitogen-activated protein kinase and many more (4)] and thereby enable computer-based analysis of complex biological systems. We observe that many models reassemble large biochemical reaction networks. They may have been semi-automatically generated using data driven approaches, for example, to construct models from metabolic networks (5, 6). Models may also prove a theory or concept, for example, to mathematically describe interactions between biological entities (7) or generic oscillatory networks of transcriptional regulators (8).

Many models are published in the Systems Biology Markup Language (SBML) (9). SBML is a well-defined file format for the exchange of models between software systems (10). It focuses on representing biological processes as sets of interactions between biological entities. A resource of openly available and reusable SBML models is BioModels (11). Release 29 of this repository contains 575 curated and semantically annotated SBML models. Further resources for reusable models are the Physiome Model Repository (12) and the JWS Online Model Database (13). The large number and size of available models require automated methods and computational tools to support researchers in identifying common phenomena; exploring sets of models; and coupling, merging or combining models. All these tasks require means to compare the characteristics of models. A variety of similarity measures have been proposed and implemented, focussing on syntactical similarity, on the evaluation of semantic annotations and on indexing the reference publications (14). When models are presented as network graphs, it will be natural to also compare them by network structure (14) and using network analysis. Methods from this field of research mostly focus on network diameter and network efficiency (15), on the topological and dynamical properties that control the behaviour of the network (16) or on the degree of tolerance against errors in scale-free networks (17). While these approaches provide key figure values for the network topology, they do not provide sufficient means to structurally characterise and compare a set of network models. Lakshmi and Meyyappan (18) state that it is possible to regard the composition of network elements by viewing network graphs as similar, if they share many common substructures. Consequentially, the problem of detecting structural similarities within network models can be defined as a frequent subgraph mining (FSM) task (19).

In this paper, we present a five-step workflow for the discovery of structural patterns in biological networks: (i) import models, (ii) export networks, (iii) create labelled graphs from networks, (iv) execute graph mining, (v) visualize and distribute patterns. The workflow implementation imports a set of SBML-encoded models in graph-representation. It then extracts all reaction networks belonging to these models. Based on the network structures converted into a standard graph format, a mining algorithm identifies frequently occurring patterns. Finally, the patterns are visualized, and their distribution among the model set is computed. We show exemplary patterns, purely structural and also incorporating annotations to the Systems Biology Ontology (SBO) (20), which were detected in curated SBML models by means of the proposed workflow. An automated retrieval of common patterns enables various types of investigations, such as ‘What are frequently used structures to represent biochemical processes?’; ‘Can we find unique patterns for certain modelling techniques (theoretical, data driven, or hybrid)?’; ‘Do frequent patterns reflect well-known motifs in Systems Biology, such as functional motifs proposed by Tyson and Novk (21)?’; ‘Can we cluster a set of models with regard to occurrences of certain patterns?’. Furthermore, the detection of reoccurring structures within a set of biochemical reaction networks is a first step towards structure-based similarity measures.

The next section provides an overview of graph mining algorithms and tools that are relevant for this work (section ‘Materials and Methods’). Afterwards, the workflow steps are described in full detail (section ‘Results’) and use cases of the output are exemplified with a set of SBML models from BioModels (section ‘Exemplary Application’). At the end of the paper, we discuss how the identification of patterns by our workflow could contribute to answering questions such as those referred to above, and we outline ideas for further investigations (section ‘Discussion’).

## Materials and methods

Data mining is a common technique for the extraction of implicit, non-obvious information from huge datasets (22). The mining of frequent patterns has its roots in the early 90s, when it had been used to examine the customers’ buying behaviour. Sales could be increased by detecting patterns in frequent combinations of bought products (23). We focus on graph-based approaches in data mining, because our models are represented by reaction networks. Approaches for identifying patterns in graphs are, for example, based on set-similarity (24), hypergraph analysis (25) or require specific types of edges and vertices, e.g. the existence of taxonomic relationships (26, 27). For this work, we chose FSM (18), which addresses the following problem: given a set of graphs, find those subgraphs within the graphs that pass a given frequency threshold (28). To decide whether a graph is embedded in another, FSM algorithms require subgraph isomorphism testing (18). This is known as an NP-complete task. Thus, FSM techniques rely on prior knowledge, heuristics and further domain-dependent strategies to improve the performance. A variety of FSM algorithms have already been implemented (19). It should be noted that most FSM algorithms are used in a domain-specific manner. For example, an FSM algorithm exists specifically for molecular databases with structures of atoms and bonds (29).

For our application domain, we decided to use gSpan (30). GSpan takes a set of graphs as input, in this case a set of reaction networks, and produces all frequent connected subgraphs according to a given frequency threshold, i.e. gSpan searches for structures that occur in at least a certain number of graphs within the set. While other algorithms supply only approximate results, gSpan fulfils our requirement for exact results. Wörlein *et al.* evaluate and compare the performance of the subgraph miners MoFa, gSpan, FFSM and Gaston (31). For this purpose, the authors developed a tool called the ‘Parallel and Sequential Mining Suite’ (ParSeMiS). ParSeMiS is based on Java and implements algorithms such as gSpan, Gaston and Dagma. In addition, Priyadarshini and Mishra (32) described a detailed approach to graph mining using the gSpan algorithm.

Current network analysis mostly focuses on network diameter and network efficiency (15), on the topological and dynamical properties that control the behaviour of the network (16), or on the degree of tolerance against errors in scale-free networks (17). These approaches provide key figure values for the network topology, but they do not detect actual patterns. On the other hand, biologists have an interest in classifying models by their function. While analysing the function of patterns requires knowledge of a domain expert, frequently occurring patterns can be determined automatically. Wong *et al.* (33) discuss the biological significance of network patterns and present several algorithms to identify such patterns. These algorithms are compared and classified. Searches for frequent patterns were already performed in the Kyoto Encyclopedia of Genes and Genomes (KEGG) (34). Hattori *et al.* (35) describe a method to compare chemical structures of the KEGG LIGAND database by identifying their common patterns. The considered chemical structures are mostly metabolic compounds. The atoms and covalent bonds are represented as graphs, where the maximum common subgraph is searched for all possible pairs of compounds. The procedure is applied to detect frequent patterns in 9383 compounds and to cluster these compounds according to their similarity. Koyutürk *et al.* (36) propose an algorithm to discover frequent patterns within a set of metabolic pathways in the KEGG PATHWAY database. The algorithm performs FSM on the metabolic pathways that are represented as directed graphs. The authors show exemplary results for detected patterns. The computational cost for the algorithm is reduced by utilizing the sparse nature of metabolic pathways and unique node labelling. The case of SBML models is more complex as their networks are not sparse and their entities are annotated with a variety of terms stemming from different ontologies.

In the field of business informatics, Li *et al.* (37) propose a method to extract occurring subgraphs in a repository of business process graphs, compute the distance between the user’s process model and the extracted patterns, and recommend a ranked list of patterns. By remodelling the process graphs to be represented uniformly, they can even find large patterns or rather the ones only contained in a few networks.

## Results

**Figure 1.**
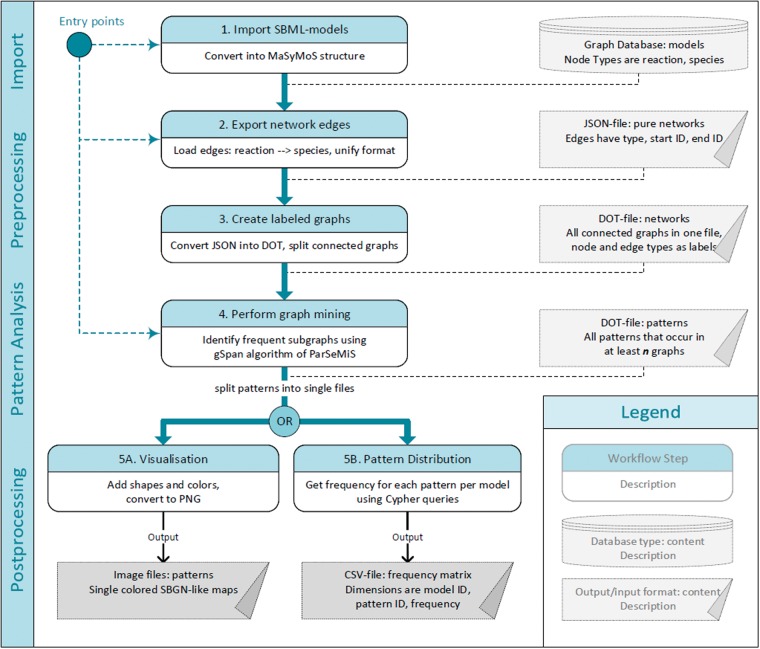
Pattern identification workflow. Numbered, oval boxes describe workflow steps. Rectangular boxes describe data produced by each step and taken as an input for the following step. Starting at multiple entry points is possible depending on the available data and format. The first step is to import models into the MaSyMoS graph database. From there the reaction networks are extracted and converted to a uniform structure. The converted reaction networks are used as an input for the subgraph mining step to identify patterns. Subsequently, two options to further process the pattern descriptions are available. On the one hand, images representing the patterns can be generated (output on the bottom left). On the other hand, patterns can be fed back to the database to create a feature matrix showing the distribution of identified patterns among the models (output on the bottom right).

We designed a five-step workflow to retrieve frequent patterns within reaction networks of SBML models (see Figure 1). To store and access models, our workflow utilizes a graph database. Network structures are extracted to detect occurring patterns by means of the FSM algorithm gSpan. The generated patterns can be visualized using glyphs compliant to the standardized Systems Biology Graphical Notation (SBGN) (38). Furthermore, the pattern distribution among all models can be computed. The workflow has different entry points, which can be chosen depending on the available data. Below, we explain the single steps in detail.

### Step 1: Import SBML models

The workflow may either be applied to models already existing in the graph database MaSyMoS (39) or to a custom set of models. The published instance of MaSyMos is shipped together with a database (https://github.com/FabienneL/BioNet-Mining/tree/master/data) containing all curated models of BioModels Release 29 (ftp://ftp.ebi.ac.uk/pub/databases/biomodels/releases/2015-04-16/). Additional SBML models can be imported into a local MaSyMoS instance. In MaSyMos, the SBML structure is mapped onto a custom graph structure, which preserves network information: the species and reactions are represented by nodes and their relations are represented as edges between them. A species can take the role of a reactant, modifier or product. Relations between species and reactions are bidirectional. Of particular importance are the relations ‘a reaction HAS participants’ and ‘a species IS participant’ in a reaction. When applying the workflow to a custom set of models, the representation of networks must be graph-based and comply with the structure available in MaSyMoS. A detailed description of the mapping is available from (39).

### Step 2: Export network edges

**Figure 2.**
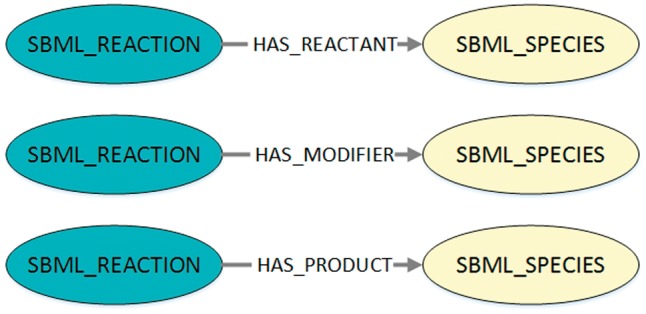
Visualization of the queried structures to export SBML reaction networks from MaSyMoS. The figure shows the three possible connections between a reaction and a species in MaSyMoS, which are reflected by different edge types. The associated cypher query in Listing 1 searches for all these structures.

Using a query interface for MaSyMoS and the query language Cypher (https://neo4j.com/developer/cypher-query-language/), our script retrieves all reaction networks of the SBML models that are present in the database. The corresponding Cypher query is shown in Listing 1 and the queried structures are visualized in Figure 2. By adapting the script, a custom model set can be used.

**Listing 1.** Cypher query to export the SBML reaction networks stored in MaSyMoS. All structures connecting reactions and species are exported. The output is a set of three-tuples consisting of the reaction’s identifier, the role type, and the species’ identifier.

MATCH (reaction: SBML_REACTION)-[edge]-> (species: SBML_SPECIES)

RETURN ID(reaction), TYPE(edge), ID(species)

**Figure 3.**
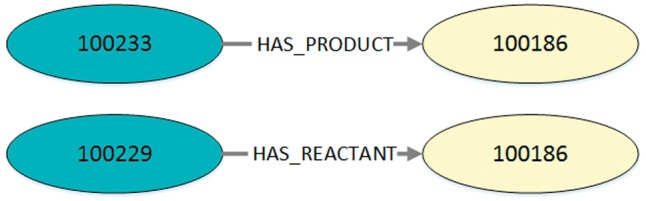
Visualization of the exemplary query output in Listing 2. Every output entry in the listing reflects an edge with its corresponding start node and end node. This figure shows two exemplary edges found in MaSyMoS that are characterized by the ID of the start node, the ID of the end node and an edge type.

We only query the nodes with their edges and do not incorporate further information, such as the associated model, publication etc. For this reason, unconnected reaction networks belonging to the same model will not further be associated with each other. The query result is a set of typed directed edges with a reaction as start node and a species as end node. Each result entry is a three-tuple containing a reaction ID, role type (reactant, modifier, product) and a species ID. The resulting set of tuples is provided as JSON output. An example is shown in Listing 2 and visualized in Figure 3.

**Listing 2.** Exemplary output (JSON) for the query in Listing 1. It contains a table with columns defining are action’s identifier, an edge type, and a species’ identifier. Consequently, the table entries are three-tuples, each representing an edge with start node, end node, and role type. In this example the IDs 100233 and 100229 represent the reactions cdc2k dephosphorylation and cdc2k phosphorylation. ID 100186 is the species cdc2k.

{

‘columns’: [‘ID(reaction)’, ‘TYPE(edge)’,    ‘ID(species)’],

‘data’:

[

  [100233, ‘HAS_PRODUCT’, 100186],

  [100229, ‘HAS_REACTANT’, 100186],

   …

]

}

### Step 3: Create labelled graphs

For later analysis, the JSON-file must first be converted into a graph representation format. We provide this information in the graph description language DOT. The associated framework Graphviz (http://www.graphviz.org/) offers manifold opportunities to process graphs by providing a collection of tools using DOT-files as input. We use a few of the tools in later steps of the workflow.

**Figure 4.**
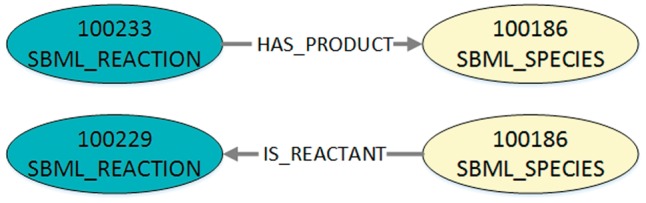
Visualization of the exemplary query output after conversion into DOT-format. The figure shows the two exemplary edges that are defined in Listing 2. In DOT-format the nodes are additionally labelled as either SBML reaction or SBML species. The edge type in the DOT-format is also defined as a label. Furthermore, the edge direction and type for the reactant role is adjusted.

To translate the JSON-file into DOT-format, we convert each three-tuple into a graph with a start node, an end node and an edge between them. The start and end node are characterized by their unique identifier and their node type (species or reaction, stored in a DOT-label). An edge is defined by the identifiers of its start and end node, its direction and its type (representing the role, stored in a DOT-label). As it is more natural for the order of nodes in the visualization that reactants and modifiers are ingoing for a reaction and products outgoing, the edge directions are adjusted. Thus, possible edge labels are IS_REACTANT, IS_MODIFIER or HAS_PRODUCT. An example for the entries resulting from the two edges of the exemplary JSON-file converted into DOT-format is shown in Listing 3 and visualized in Figure 4.

**Listing 3.** Exemplary DOT-format after converting the output (JSON) shown in Listing 2. This transitory format defines one digraph (directed graph) containing all exported nodes and edges. For each three-tuple from Listing 2, two nodes and one edge are defined. Consequently, the created digraph may contain nodes multiple times (shown here, the node with ID 100186) because one node can be part of several edges. In this example the IDs 100233 and 100229 represent reactions cdc2k dephosphorylation and cdc2k phosphorylation. ID 100186 is the species cdc2k.

digraph {

 100233 [label = SBML_Reaction];

 100186 [label = SBML_Species];

 100233 -> 100186 [label = HAS_PRODUCT]

 100229 [label = SBML_Reaction];

 100186 [label = SBML_Species];

 100186 -> 100229 [label = IS_REACTANT];

 …

}

**Figure 5.**
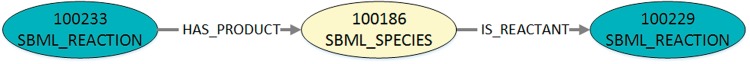
Visualization of the exemplary DOT-output after post-processing shown in Listing 4. After splitting the one digraph into its connected digraphs, there are no redundant nodes left. The figure shows the two exemplary edges, but now they are represented as a connected network, because they share their species node.

The resulting DOT-file defines one graph with nodes and edges from all reaction networks. It should be noted that the file may contain nodes multiple times, because we create one entry for a node each time it occurs as a start or end node in an edge. Consequently, we bundle all connected nodes with their corresponding edges as one graph each and eliminate redundant nodes. This is possible by means of a Graphviz tool to split a graph into its connected components. Then, each connected reaction network represents its own graph in the new DOT-file and has no redundant nodes anymore. It should be noted that SBML allows for the definition of interactions between entities as rules instead of reactions. As MaSyMoS does not build explicit species-to-species connections based on the assignment rules, we have to neglect rules and only consider explicitly connected entities. It is thus possible to have more graphs defined in the DOT-file than models used as input for the workflow. Such unconnected reaction networks belonging to the same model will not further be associated with each other. Listing 4 shows the final DOT-format for our example, and Figure 5 visualizes the connected reaction network.

**Listing 4.** Exemplary DOT-format necessary for the subgraph mining process created by splitting the digraph from Listing 3. Here, each connected reaction network is represented by one digraph and has no redundant nodes left. In this example the IDs 100233 and 100229 represent reactions cdc2k dephosphorylation and cdc2k phosphorylation. ID 100186 is the species cdc2k.

# connected reaction network 1

digraph {

 100233 [label = SBML_Reaction];

 100186 [label = SBML_Species];

 100233 -> 100186 [label = HAS_PRODUCT];

 100229 [label = SBML_Reaction];

 100186 -> 100229 [label = IS_REACTANT];

 …

}

# connected reaction network 2

digraph {

 …

}

…

### Step 4: Perform graph mining

The created DOT-file is the input for the graph mining and the basis for finding frequent patterns in the set of reaction networks. The frequency of patterns is equal to the number of reaction networks, in which a pattern occurs. Each pattern is thus counted only once for each network, even if it occurs multiple times in a model’s reaction network. We use the implementation of the gSpan algorithm in the software tool ParSeMiS to calculate frequencies: Given the user-specified values min (minimum frequency) and max (maximum frequency), the mining finds all subgraphs that occur in at least min and at most max of the graphs. We call these subgraphs frequent patterns. It does not matter for the algorithm, how often a pattern occurs within one graph, only the number of graphs is relevant. Consequently, the frequencies are values between one and the total number of graphs in the DOT-file. As already mentioned, one model may have several unconnected reaction graphs. Therefore, the number of defined graphs can be higher than the number of models used as input.

**Figure 6.**
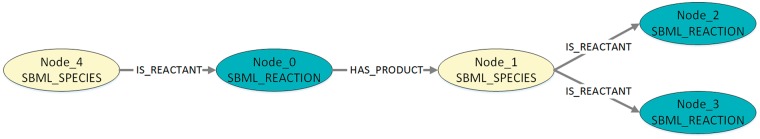
Visualization of the exemplary pattern mining result in Listing 5. The figure shows a pattern with two species and three reactions.

As gSpan is an extension based algorithm, it starts with frequent nodes and iteratively adds one of each possible edges. The result of the subgraph mining is one DOT-file containing all subgraphs having a frequency within the given interval. Thus, the DOT-file not only contains the largest resulting patterns, but also each subgraph these are based on. Every graph in the DOT-file is numbered according to the extension-process. If one subgraph is an extension of another, they have the same number. For each pattern in the DOT-file the frequency of its occurrence and the names of the corresponding models are attached as a comment. An exemplary output is shown in Listing 5 and visualized in Figure 6.

**Listing 5** **.** Exemplary pattern mining results (DOT-format). The output contains for each detected pattern one directed graph. The digraph numbering, denoted by ‘…’, can be discarded. First, all nodes are defined, starting with ‘Node_0’. Second, the edges are defined. Following a digraph’s definition, a comment (introduced by #) contains the number of graphs in which the described pattern occurs. The following square brackets can also be discarded.

digraph ‘560’ {

 Node_0 [label=‘SBML_REACTION’];

 Node_1 [label=‘SBML_SPECIES’];

 Node_2 [label=‘SBML_REACTION’];

 Node_3 [label=‘SBML_REACTION’];

 Node_4 [label=‘SBML_SPECIES’];

 Node_0 -> Node_1 [label=‘HAS_PRODUCT’];

 Node_1 -> Node_2 [label=‘IS_REACTANT’];

 Node_1 -> Node_3 [label=‘IS_REACTANT’];

 Node_4 -> Node_0 [label=‘IS_REACTANT’];

 }#=> 398[, …,]

digraph ‘560’ {

 …

}# => 436[, …,]

…

To find those subgraphs within the graphs that pass a given frequency threshold requires subgraph isomorphism testing (18). Because this is known as an NP-complete task (28), the minimum frequency must be chosen carefully. If the minimum frequency is set too low, the computation will not succeed due to capacity limitations (memory or time).

### Step 5: Pattern post-processing

The generated patterns may be used in various ways. Here, we illustrate two possible options to further process them: the first option is the visualization; the second option is the computation of frequencies for each pattern per model. In both cases, the DOT-file is split into multiple DOT-files each containing one pattern. The name of a DOT-file comprises the pattern’s frequency and an identifier. The identifier is used to distinguish between several patterns occurring with the same frequency.

### Step 5A: Visualization

The visualization follows the standardized SBGN. Thus, node and edge labels are expressed by the visualized shape suggested by SBGN and textual display of node and edge labels is disregarded. The contour, fill colour and size of nodes, and the stroke width, direction, arrowhead and size of edges are set. For each DOT-file an image-file is rendered. The standard image-format is PNG, but other formats such as PDF are supported by the DOT framework.

### Step 5B: Pattern distribution

**Figure 7.**
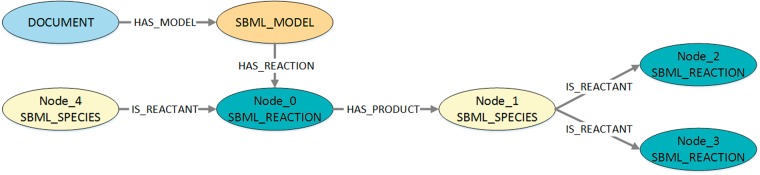
Visualization of the structure queried in Listing 6. The figure shows the structure that is queried to get information about the distribution of the pattern shown in Listing 5. To retrieve the corresponding model identifiers and model names, the queried structure also contains the associated model and document nodes.

To compute the frequencies of patterns per model, a Cypher query is generated for each DOT-file. An example is shown in Listing 6 and visualized in Figure 7. It describes the graph structure that is queried to get information about the distribution of the pattern shown in Listing 5. Further, a restriction is added that nodes are not allowed to be equal. Subsequently, the queries are executed on the MaSyMoS database and the results stored as JSON-files, listing all distinct model IDs, the model names that contain the pattern, and how often a pattern is present in each of those models. All JSON-files are then processed to create a CSV-file representing a frequency matrix. Here, the first two columns specify the model. The following columns define the patterns. Each row contains a model ID in the first column, a model name in the second column, and the frequency of each pattern in the following columns. Thus, each row can be seen as a feature vector for one model.

**Listing 6.** Exemplary Cypher code to query MaSyMoS for the distribution of a certain pattern. The exemplary pattern here represents a chain with two reaction nodes and one species node. The species takes a role as product in the first reaction and a role as reactant in the second reaction. Furthermore, it is defined that nodes are not allowed to be equal. The result is a set of three-tuples, each containing a model identifier, a model name (stored as attribute in the associated document), and the number of occurrences of the pattern. The checks for inequality in the WHERE clause are necessary to exclude cycles.

MATCH (m: SBML_MODEL)–>(d: DOCUMENT), m-[HAS_REACTION]->Node_0,

   Node_0-[: HAS_PRODUCT]->Node_1, Node_1-[: IS_REACTANT]->Node_2,

   Node_1-[: IS_REACTANT]->Node_3, Node_4-[: IS_REACTANT]->Node_0,

WHERE Node_0<>Node_1 AND Node_0<>Node_2 AND Node_0<>Node_3 AND Node_0<>Node_4 AND Node_1<>Node_2 AND Node_1<>Node_3

 AND Node_1<>Node_4 AND Node_2<>Node_3 AND Node_2<>Node_4 AND Node_3<>Node_4

RETURN DISTINCT ID(m), d.FILENAME, COUNT(Node_0)

AS sum ORDER BY sum DESC

## Exemplary application

Using the aforementioned combination of tools and methods, we exemplarily analysed two datasets on a cluster node (180 GB RAM, 16 Intel(R) Xeon(R) CPU X5650 @ 2.67 GHz).

### Dataset

For the pattern detection, we incorporated publicly available models from BioModels. The stored reaction networks are encoded in SBML. BioModels contains two types of models: curated and non-curated. We here chose only models from the curated branch as those models can be expected to accurately represent the work described in the reference publication. Furthermore, curated models are syntactically and semantically validated and annotated with ontology terms, and they comply with the MIRIAM standard (40). Specifically, we analysed SBML models from two different releases of BioModels. Release 1 (in the following referred to as R1) is the first release of the repository. It contains 30 curated models. Release 29 (in the following referred to as R29) is one of the latest releases. It contains 575 curated models. We chose these two releases to take the evolution of BioModels into account. As we decided to perform subgraph analysis with an FSM algorithm, we translated the biological reaction network into a graph representation using the MaSyMoS database. For the reaction network, the MaSyMoS graph structure distinguishes two types of nodes (i.e. labelled *species* and *reaction*) and three types of edges (labelled *is_reactant*, *has_product* and *is_modifier*).

### Quantitative analysis

First, we performed a key figure analysis to calculate the quantities of node types and edges in the networks. In our dataset, 557 out of 575 models in R29 contain species, and 499 models contain reactions. The remaining models only define rules, but do not form a network. The dataset contains a total of 18 852 reaction nodes and 16 843 species nodes.

**Figure 8.**
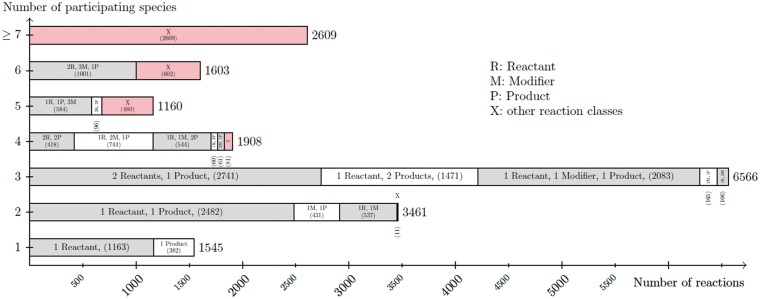
Listing of the node degree for reaction nodes in the dataset R29 of curated models in BioModels Database. For each number of species (from 1 to 6, and more then 6) participating in a reaction, the figure lists the number of reaction nodes identified with a particular combination of its species relations (reaction class). The figure sums up smaller reaction classes displayed by X.

Dataset R1 contains only 30 curated models. These models contain a total of 736 reactions and 425 species. The big difference in numbers between R1 and R29 are due to the rapid growth of models, as previously reported (2). On average, a model from R29 has 30.2 species and 37.7 reactions. In R1, a model has 14.6 species and 25.4 reactions on average. For both datasets most models contain 3-11 species. In addition, most models have 3–12 reactions. However, there are a few outliers with >100 reactions and species. Figure 8 shows the correlation between species (and their respective role as reactants, products and modifier) and reactions. As the figure states, most reactions have two or three participating species. The most frequently encoded reaction has two species as reactants and one species as product. The second most frequently encoded reaction has one species as reactant and one as product.

### Exemplary patterns

We identified a subset of patterns shared by at least a certain number of models. For data set R29, we were able to identify 37 patterns in total. Each identified pattern is shared by at least 350 out of 575 models. For the much smaller dataset R1, we identified 190 patterns. Here, each pattern is shared by at least 20 out of 30 models. The visualizations of all exemplary patterns are available from the project repository (https://github.com/FabienneL/BioNet-Mining). For R29, the identified patterns contain between 1 and 6 entities (species or reactions) whereas patterns for R1 contain between 1 and 11 entities. It was not possible to further scale down the number of models that share a pattern due to memory limitations.

#### Common types of reactions

From the quantitative analysis and the statistics shown in Figure 8, we expected to see patterns having one reaction and three species (participating as product, reactant or modifier). Surprisingly, the pattern identification shows that no such patterns are shared by at least 350 models in R29 or by at least 20 models in R1, respectively. Subsequently, we searched for expected structures having one reaction and three species in the MaSyMoS database. The specific combination of two reactants as a reaction’s input and one product as a reaction’s output only occurs in 314 models, despite being the most frequently encoded reaction class according to Figure 8. Same holds for all other possible reaction classes with three species for R29 and R1, respectively. One can conclude that such types of reactions are often used, but are not equally distributed across models. Instead, many models contain patterns with a central species node that participates in several reactions. Furthermore, the retrieved patterns mostly describe chains and often contain a single branch.

#### Species as a reaction modifier

**Figure 9.**
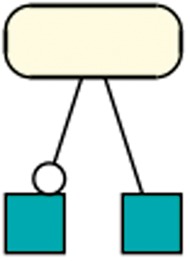
Species as modifier: This pattern occurred in 351 models of dataset R29 and shows a species taking part in one reaction as a modifier and as a reactant in a different reaction. A rectangular SBGN PD glyph with rounded corners states an entity, the SBGN PD square box indicates a reaction. The modifier role is indicated by a circle attached to the black line, the reactant role is indicated by a straight line (41).

Generally, species in R29 most often take part in a reaction as a modifier (33 209 times), and less as a product (23 630) or reactant (25 595). However, only 4 out of the 37 retrieved patterns (R29) contain species that act as a modifier. One of those four patterns is shown in Figure 9. A further investigation reveals the unequal distribution of modifiers among the models. Ten models together count for 20 620 modifier usages. Among those ten models, five models are derivations of the aforementioned semi-automatically created models of metabolic networks (6).

#### Motifs

Biologists have an interest in classifying models by their function. Tyson and Novk (21), for example, were interested in the mechanisms of information processing. They showed that complex networks could be decomposed into simple patterns, each fulfilling specific functions within a cell. These patterns were postulated as common motifs in biochemical reaction networks. It remains an open question how and how frequently these motifs are encoded in a model. Figure 11 shows the network motifs that were postulated by Tyson and Novk (21). The structure of Motifs 3-5 can be represented as graphs, in which two species and two reactions form a cycle. Such motifs can encode, for example, the production and degradation of a protein, or positive or negative feedbacks.

**Figure 10.**
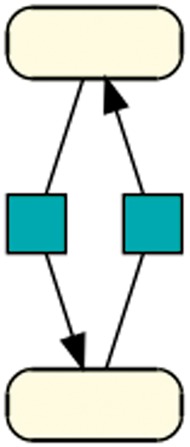
Cycle: This pattern shows the smallest biologically meaningful cycle with 2 species and 2 reactions. The directed black arrow defines a species as a reaction’s product (41). This pattern is contained in 330 models of dataset R29 and in 25 models of dataset R1.

While analysing the function of patterns requires knowledge of a domain expert, frequently occurring patterns can be determined automatically. Using our workflow, we identified one pattern that represents the structure of Motifs 3–5; it occurs in 26 models of R1 (shown in Figure 10). However, this pattern is not among the 37 patterns retrieved using dataset R29. A subsequent query in MaSyMoS for the exact pattern reveals that the structure indeed only occurs in 342 models. Surprisingly, the query retrieved >45 000 occurrences of this cycle in R29. To investigate further, we ordered the results by model. Again, the answer is the distribution of the pattern: two models by Stanford *et al.* (5) (generated semi-automatically) count for approximately 10 000 cycles each. Together with our observations regarding the usage of species as modifiers in reactions, we can assume that semi-automatically generated models have a distinguishable network structure. BioModels contains two prominent examples of such models (5, 6).

#### Pattern identification (semantics-aware)

**Figure 11.**
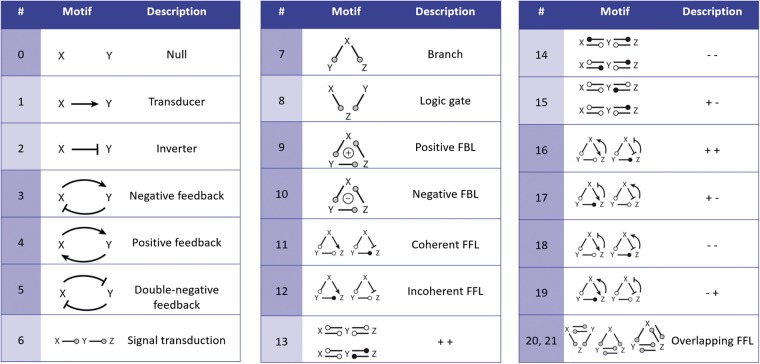
Functional motifs postulated by Tyson and Novk (21): A grey circle in a motif indicates an interaction that may be either + or −. All white circles in a motif must have the same sign, either + or −, and they must be of opposite sign to any black circle in the same motif. FFL denotes a feed-forward loop and FBL denotes a feedback loop. We grouped these motifs by structure. For example, Motifs 3–5 are grouped as they are all cycles of two species and two reactions. An analogous group is built by Motifs 9–12. The groups are depicted by alternating colours.

Our workflow currently does not consider semantic annotations and thus cannot provide information about the intended semantics of reactions and species. Consequently, we cannot distinguish all of the postulated motifs. For example, the pattern describing a simple cycle (*cmp.*Figure 10) could be corresponding to Motif 3, Motif 4 or Motif 5 in Figure 11.

**Figure 12.**
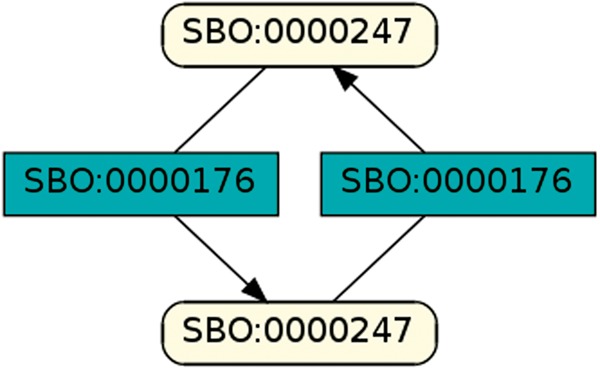
Simple chemical and biochemical reaction.

**Figure 13.**
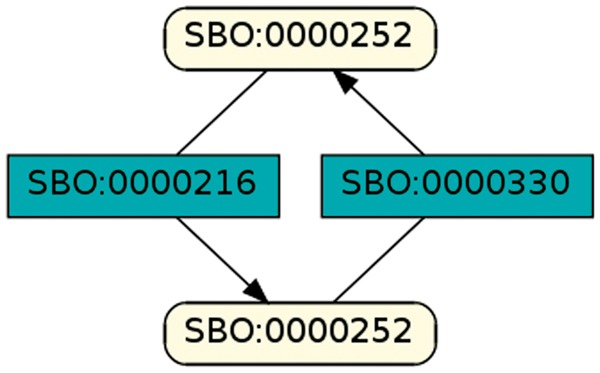
Phosphorylation and de-phosphorylation of a polypeptide chain.

To regard semantics, we adapted Step 2 of the described workflow. The network extraction was refined to additionally receive the SBO-annotations for the species and reactions. SBO terms define the semantics of model components, including their physical type, their biological role in a reaction, or the type of a process (20). SBO-annotations found in SBML models reflect the biological role of a species or reaction. Two downsides of incorporating SBO-annotations have to be considered: first, only 116 out of 575 (R29) models have reaction networks annotated with SBO terms. Second, as Alm *et al.* (42) state, the specificity of SBO-annotations varies among models. Taken together, the remaining reaction networks are less complex, allowing us to retrieve 176 patterns contained by at least 12 out of 116 valid models. Structure-wise, Figures 12 and 13 are equivalent to Figure 10, but they now include semantics, i.e. the role of each participating species and reaction. Figure 13 is an identified pattern that describes circular reactions (SBO:0000176) between simple chemicals (SBO:0000247) and Figure 13 describes the phosphorylation (SBO:0000216) or de-phosphorylation (SBO:0000330) of two polypeptide chains (SBO:0000252).

**Figure 14.**
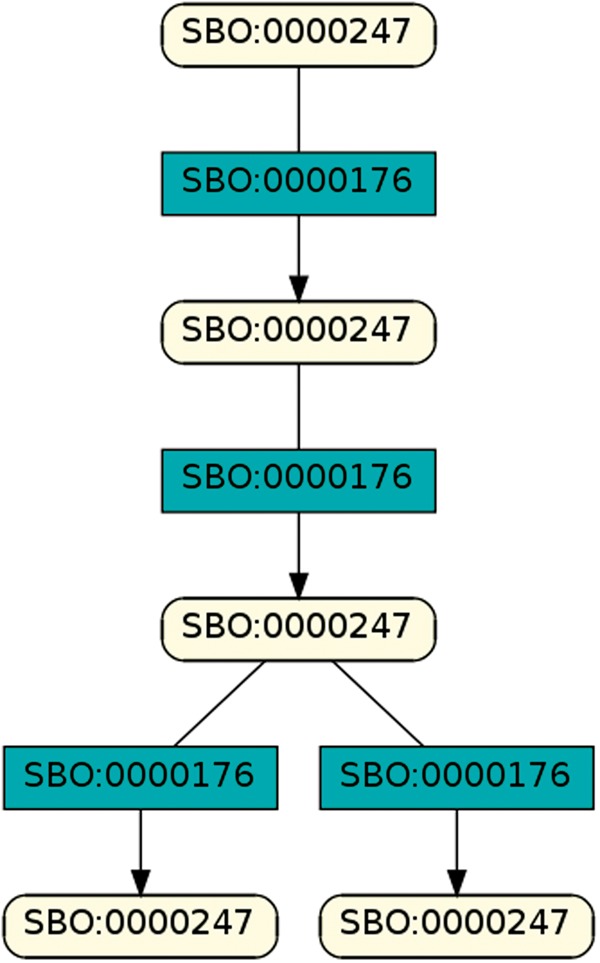
Exemplary pattern, a combination of simple chemical and biochemical reaction.

**Figure 15.**
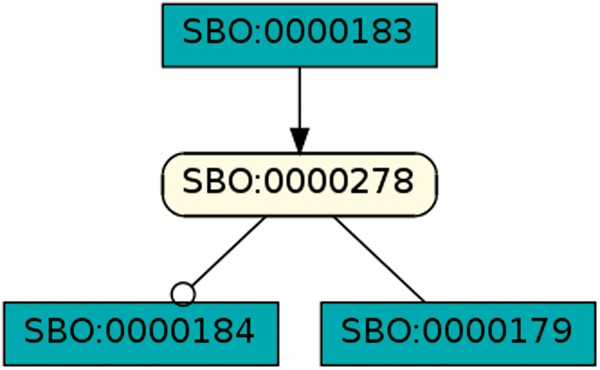
Transcription, translation and degradation of messenger RNA.

A brief analysis of all retrieved SBO-based patterns reveals structures similar to Figure 14. In fact, all but one pattern with at least four entities contain a combination of simple chemical (SBO:0000247), biochemical reaction (SBO:0000176), or phosphorylation (SBO:0000216), de-phosphorylation (SBO:0000330) and polypeptide chain (SBO:0000216). The one outsider pattern encodes the transcription, translation and degradation of messenger RNA (*cmp.*Figure 15).

Not all models are annotated with SBO terms and mostly the annotation level is abstract (42). To consider biological semantics in more detail, a pre-processing using annotation propagation (43) or inferring math dependency maps (44) can be applied. Our approach, however, is solely based on explicitly encoded information.

#### Feature matrix

Current approaches for model clustering only incorporate semantic annotation and meta-information (42, 45). Our work is a first step towards creating structural similarity measures for biological models. We hypothesise that these similarity scores can help to distinguish models, for example, to classify them by a certain modelling technique (theoretical, data driven or hybrid). Having identified patterns at hand, it is easy to generate a vector for each model holding the number of occurrences for each pattern within a model. Using the approach of term frequency and inverse document frequency with a vector space model, well studied in the field of information retrieval, one can draw conclusions about the similarity of models based on shared patterns. However, it is not feasible to use all identified patterns for such a model comparison. Instead, patterns should be weighted according to their biological significance. It also seems promising to incorporate information about the uniqueness of a pattern, i.e. does the pattern contain other identified patterns itself. Such an analysis would lead to an approach similar to eTVSM (46).

## Discussion

In this paper, we present a five-step workflow for the retrieval of frequent patterns in SBML models. The work is a first step towards a new structural analysis of biological networks. We utilized the proposed workflow to exemplarily analyse reaction networks from curated SBML models (release 1 and release 29 of BioModels). For example, we searched for frequently used structures that represent biochemical processes. Therefore, we compared structural patterns resulting from the workflow with key figure values from a quantitative analysis. While the retrieved patterns reflect structures occurring in a high number of models, the quantitative analysis reveals how often certain structures occur in general. We found that although one reaction with three participating species is a frequently modelled structure, no such pattern was retrieved in our test run. Instead, patterns were retrieved that contain a species as a centre node participating in several reactions. A remaining question is, whether frequent structures reflect biological phenomena, modelling phenomena or both. Thinking along these lines, we hypothesise that the higher number of modifiers and cycles that we identified in semi-automatically generated models hint at a distinguishable network structure for certain modelling techniques. In future investigations, semi-automatically generated models could be used as input for the workflow to search for unique patterns in this model set.

Moreover, the presented workflow could be used to test hypotheses about reoccurring patterns in domain-specific model sets. In this context, the following questions could be examined: ‘Is it possible to infer the biological domain of models by occurrence of patterns in their networks?’; ‘What are characteristic patterns in the class of cell cycle models?’; ‘Do models from different biological domains (e.g. cell cycle, apoptosis, transport, metabolism) share patterns?’

An automated retrieval of common patterns is also a first step towards creating structure-based similarity measures for biological models. A number of approaches exist to compare models based on the encoding format, the XML tags, or semantic annotations. It is, for example, interesting to study models regarding function, structure and behaviour (47); regarding their temporal evolution (48, 49); or regarding their dynamics (50). However, as Lakshmi and Meyyappan (18) state, network graphs can also be considered similar, if they share many common substructures. Thus, patterns retrieved from our workflow can be used as basis for calculating structural similarity. By generating a vector for each model holding the number of occurrences for certain patterns within the model, it will be possible to apply established vector similarity measures. Based on the calculated similarities, models could then be classified or clustered regarding common patterns. The suggested structural approach could also extend existing methods for grouping biological models. We hypothesise that this can help to better distinguish models. The consideration of patterns will enable search for models that share similar structures, improve the mapping of similar models onto each other (51), and lead to recommender systems that support the modelling process. In addition to already existing similarity measures (14), this work will impact the reuse and reproducibility of scientific modelling results.

Our five-step workflow not only discovers structural patterns. It also allows to calculate the distribution of patterns among models and to visualize retrieved patterns. This visualization follows the SBGN standard. By providing a standards-compliant visualization, the patterns are more easily comparable to other works, for example to the already existing SBGN bricks (52). The workflow can be adapted and extended. It is possible to adapt the pre-processing steps to enable pattern detection in CellML-encoded models, or even in other model representation formats. The pre-processing could also be adapted to better incorporate semantic information, such as the knowledge about mathematical concepts encoded in SBO terms.

In the future, we will incorporate more information about the role of a reaction (e.g. promoter or inhibitor). The use of annotations, specifically from SBO, will enable us to identify motifs more precisely. SBO provides terms for the functional role of a species or reaction but SBO terms used for model annotation are mostly abstract. For example, a species can simply be annotated as a ‘simple chemical’ (SBO:0000247). Most species and reactions in our datasets contain such annotations, but some networks are still not annotated. The consideration of annotation will also lower the computational costs for the search for sub-models, because valuable semantic knowledge can be incorporated to reduce the number of potential alignments.

## Conclusion

The increasing number of published models and the growing size of encoded reaction networks demand automated methods for model analysis. Studying biochemical reaction networks, one is mostly interested in biological systems that share similar reactions and mechanisms. These substructures in networks are essential for researchers to determine reoccurring parts in models, or to characterise typical sub-modules. This knowledge can then help to identify common biological phenomena, to explore sets of models, and to couple, merge, or combine models. Thus, pattern detection and identification of motifs are necessary tasks of scientific interest.

In this paper, we present a workflow that addresses the problem of obtaining common patterns in SBML-encoded models by applying an FSM algorithm. Our workflow implementation loads a custom set of SBML models into a graph database and delivers information about frequent patterns in that set of models. For the pattern detection it uses a Java-based gSpan implementation. Identified patterns can be fed back into the graph database to retrieve further information, for example, about the pattern distribution. The presented workflow is openly available and can be adapted to other model encoding formats. It can also be extended to support further types of pattern analysis. When being integrated with available model repositories, information retrieved from our workflow can improve model search, comparison, and provenance.
